# Thermal influences on spontaneous rock dome exfoliation

**DOI:** 10.1038/s41467-017-02728-1

**Published:** 2018-02-22

**Authors:** Brian D. Collins, Greg M. Stock, Martha-Cary Eppes, Scott W. Lewis, Skye C. Corbett, Joel B. Smith

**Affiliations:** 1 Landslide Hazards Program, U.S. Geological Survey, 345 Middlefield Road, MS973, Menlo Park, CA 94025 USA; 2 National Park Service, Yosemite National Park, 5083 Foresta Road, Box 700, El Portal, CA 95318 USA; 3 Department of Geography and Earth Sciences, University of North Carolina - Charlotte, 9201 University City Blvd, Charlotte, NC 28223 USA; 4 Condor Earth Technologies, Inc., 21663 Brian Lane, Sonora, CA 95370 USA; 5 Landslide Hazards Program, U.S. Geological Survey, Box 25046, MS966, Denver, CO 80225 USA

## Abstract

Rock domes, with their onion-skin layers of exfoliation sheets, are among the most captivating landforms on Earth. Long recognized as integral in shaping domes, the exact mechanism(s) by which exfoliation occurs remains enigmatic, mainly due to the lack of direct observations of natural events. In August 2014, during the hottest days of summer, a granitic dome in California, USA, spontaneously exfoliated; witnesses observed extensive cracking, including a ~8000 kg sheet popping into the air. Subsequent exfoliation episodes during the following two summers were recorded by instrumentation that captured—for the first time—exfoliation deformation and stress conditions. Here we show that thermal cycling and cumulative dome surface heating can induce subcritical cracking that culminates in seemingly spontaneous exfoliation. Our results indicate that thermal stresses—largely discounted in dome formation literature—can play a key role in triggering exfoliation and therefore may be an important control for shaping domes worldwide.

## Abstract (web summary)

Thermal triggering of rock exfoliation has long been discounted as relevant to the evolution of rock domes. Here, the authors documented and measured recent fracturing events in California, USA to show that hot summer periods can lead to thermal stresses and cause seemingly spontaneous rock exfoliation.

## Introduction

Exfoliation (here referring to ≥10 m^2^ scale, surface (sub)parallel rock fracture) and its resultant landforms, especially rock domes (e.g., Yosemite’s Half Dome and Rio de Janeiro’s Sugarloaf Mountain), have attracted scientific attention for more than a century^1–11^. Exfoliation has generally been attributed to several mechanisms, including overburden removal and elastic rebound^2,5^, tectonic compressive stress^3,6^, and topographic curvature under surface-parallel compression^12,13^. Existing data, however, come from observations of static landscapes where exfoliation sheets (referring to the detached slabs of rock produced by exfoliation) and domes reside as relict features^2,6^. For example, curious tent-like structures—common on domes—have only qualitatively been explained as by-products of compression^9,14,15^. Yet, as an example of rock fracture, exfoliation is perplexing and raises questions concerning the temporal scale over which it occurs. Do exfoliation sheets slowly weather and peel away from the parent rock dome, or do they pop and fracture energetically? Are they legacy features of exhumation, or is their formation ongoing and driven by other forcing mechanisms? And what stresses must be involved to fracture rock in this way?

Recent analytical efforts using fracture mechanics theory^11–13,16^ have begun to quantify the stresses that likely arise during exfoliation. These and other studies^17–20^ focus on subcritical crack propagation, whereby stress magnitudes that are lower than the rock’s critical strength lead to slow, stable cracking over geologic time, and now provide an overall framework for exfoliation mechanics. Although recent rock weathering and exfoliation studies also employ fracture mechanics theory to implicate low stress conditions in substantial cracking over long time scales^20,21^, field studies of actual events that link models with natural conditions are lacking. This is primarily because exfoliation is rarely observed directly. Only in anthropogenic settings such as quarries and underground mines^5,22–27^ have real-time observations been made of the dynamic (critical fracture) process of exfoliation and subsequent sheet detachment, and even these, with a few minor exceptions^23^, lack the quantitative information needed to understand why, when, and how exfoliation occurs at the Earth’s surface.

Here we quantitatively document repeated spontaneous exfoliation of a granitic dome that was not subject to any direct anthropogenic forcing (e.g., not induced by excavation). Twain Harte Dome in the Sierra Nevada foothills of northern California fractured a total of eight times during the summers (June through September) of 2014, 2015, and 2016. Witnesses directly observed (e.g., Supplementary Movie 1) the energetic (i.e., critical and explosive) fracturing in all but three of the events. One event caused leakage through an 11-m-tall concrete dam founded on the dome, and a flash-flood warning ensued, underscoring the importance of understanding exfoliation in locations that commonly support infrastructure^28^.

Along with observer-based data, we use instrumental measurements (crackmeters and extensometers for rock deformation, longitudinal rockbolts for rock uplift force, acoustic emission sensors for microcracking, and rock and air temperature and relative humidity sensors) to investigate the origin of the exfoliation events at Twain Harte Dome. Some of the instrumentation captured fracture deformation and sheet stress behavior data of the active part of the dome during several of the exfoliation events. Using these data, we show that thermally driven stress—combined with long-term thermally driven subcritical cracking—was the likely trigger for exfoliation. This finding runs contrary to the conventional wisdom that thermal processes have at most only a minor role in exfoliation processes^5,6,29,30^. Our study thus sheds light on a likely mechanism by which exfoliation sheet and dome formation occur in landscapes and environments worldwide, ranging from Brazil^31^ to Finland^32^.

## Results

### Real-time exfoliation observations

**Fig. 1:**
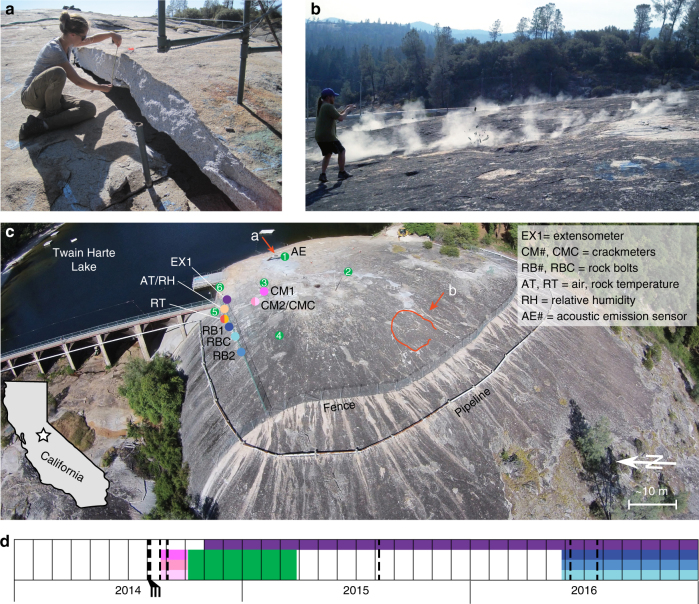
Twain Harte Dome California and exfoliation events. **a** During the summer of 2014, spontaneous exfoliation caused 30-cm-thick exfoliation sheets to fracture and tent upwards. **b** Close-range video captured the explosive nature of one instance of exfoliation (full video available in Supplementary Movie 1). Fragments of rupturing rock are visible in the center of the image and rock dust emanates from the fracture boundary. **c** Overall dome is 100 m by 350 m with maximum vertical relief of 75 m to the west. Summit area (shown here) is symmetrical with 50 m radius and increases in curvature from 0.007 m^−1^ at the summit to 0.027 m^−1^ to the northwest. White streaking on rock results from snowmelt runoff collected at the base of a chain-link fence. Deformation (EX1, CM#, CMC), uplift force (RB#, RBC), air and rock temperature (AT, RT), relative humidity (RH), and acoustic emission (AE, 1–6) instruments monitored ongoing fracture. Arrows indicate view direction for **a** and **b**; red outline shows exfoliation area captured in **b**. Inset map shows location of study area in northern California, USA. **d** Timeline of deformation and acoustic monitoring instrumentation installation, colored as shown by symbols in **c**. Black dashed vertical lines are fracturing events. Image in **c** is © Robert J. Perry, 2015 and used with permission

**Fig. 2:**
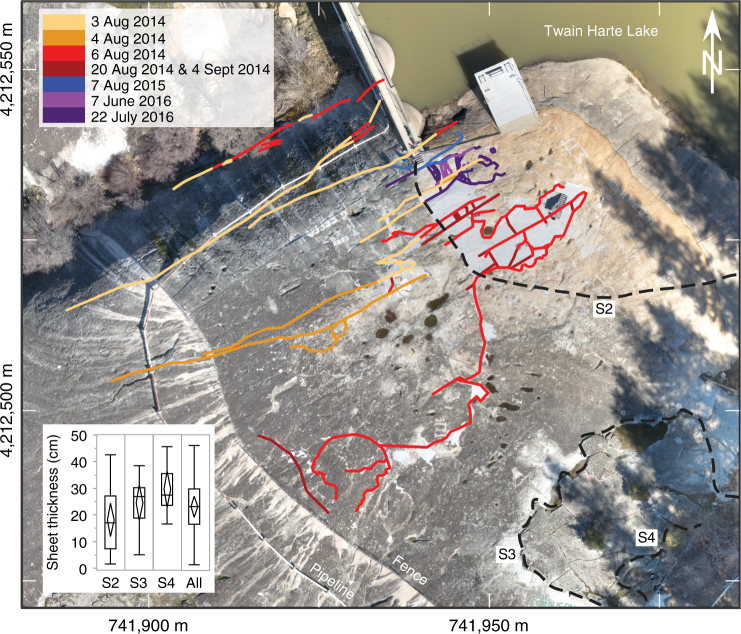
Fracture mapping of recent and older exfoliation at Twain Harte Dome. Fractures (both surface-parallel and perpendicular) formed during events of 2014–2016—colored by date—indicate exfoliation progressed first to the south and then to the northeast. Mean thickness (inset), measured along transects (dashed lines—S3, S4), of past exfoliation sheets is slightly larger than the mean thickness of newly exposed sheets on the dome surface (S2), reflecting that thinner sheets formed during recent exfoliation and have eroded from older generations. Note that  the top of sheet S1 (not labeled) is formed by the newly exposed exfoliation surface; its thickness across a transect cannot be easily measured.  Base image (NAD83, UTM Zone 10 grid shown) created from UAS (drone) orthophotography as part of this study. Inset box-and-whisker plot elements: horizontal line = median; diamond = mean (center) and 95% confidence interval; box = 25th to 75th quantiles; whiskers = all data range

**Fig. 3:**
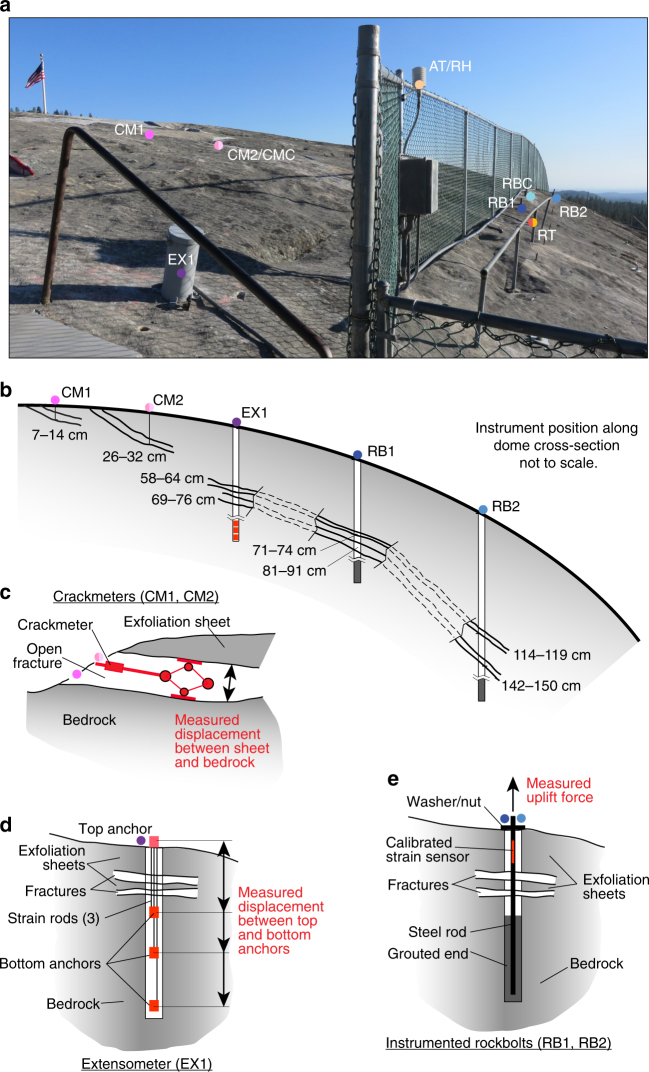
Exfoliation dome instrumentation layout and schematic diagrams of deformation and force monitoring equipment. **a** A suite of instrumentation is deployed across the most active area of exfoliation. Symbols, instrumentation labels, and scale are as described in Fig. 1. **b** Instrument installations intersect several exfoliation sheet fractures at depth depending on their position across the dome. Fractures are open across the annotated depths and are to scale. Note that instrument positions along the cross-section are not to scale (i.e., the instrumentation is not in-plane as shown; see Fig. 1 and **a** for true spatial layout). Crackmeters, extensometers and rockbolts measure longitudinal strain, which is converted to deformations and force through linear elastic constants specific to the geometry and design of each instrument. **c** Crackmeters measure exfoliation sheet fracture aperture width by installation directly within fractures. **d** Extensometers measure exfoliation sheet fracture aperture width by installation across fractures through anchoring on either side of a fracture. In our extensometer installation, three instruments provide redundant measurements across the fractures. **e** Rockbolts measure exfoliation sheet uplift force generated along a steel rod that is grouted and fixed in bedrock at one end, and tightened to the surface of the deforming exfoliation sheet at the other end. Forces are converted to stresses through approximations of exfoliation sheet attachment geometry. See Methods for additional details for instrumentation installations

Twain Harte Dome shares broad characteristics with the hundreds of other granitic exfoliation domes found in California, and elsewhere. Located on the western slope of the Sierra Nevada at an elevation of 1080 m (above mean sea level), the dome (Fig. 1) was not glaciated during the last glacial maximum^33,34^. Spontaneous popping noises from Twain Harte Dome commenced in early August 2014, with fracture formation and propagation on its north side. Several exfoliation events occurred over the next few days (Table 1), but with differing locations (Fig. 2) and associated energy. On 6 August 2014, explosive fracturing thrust a 2 m by 5 m by 0.3 m sheet of granodiorite up to 44 cm upward, forming a tent structure (Fig. 1a). Rock fracture then continued for about 20 min, eventually shifting to the southwest with rock fragments thrown more than 4 m upwards (Fig. 1b, see also Supplementary Movie 1 and hyperlinks in Table 1). Two other exfoliation events occurred over the next month, but with lesser energy (i.e., smaller area of fracturing, Fig. 2) compared to earlier events. Our crackmeters (Fig. 3) and acoustic emissions (AE) monitoring (Fig. 1c), installed in late August and early October 2014, respectively, captured some of the post-exfoliation behavior.

**Table 1:** Event sequence at Twain Harte Dome from 2014 through 2016

Date (and time, when known)	Event description	Source
3 August 2014, 08:26 (PST)	1st exfoliation event	C. Doty YouTube^a^ & personal communication (8 Sept. 2014)
3 August 2014	Begin reservoir draining	
4 August 2014	End reservoir draining	D. Wyckoff personal communication (22 Sept. 2014)
4 August 2014, 09:34 (PST)	2nd exfoliation event	C. Doty YouTube^b^ & personal communication (8 Sept. 2014)
6 August 2014, 16:04 (PST)	3rd exfoliation event	C. Doty YouTube^c^ and personal communication (8 Sept. 2014), S. Lewis video^d^
20 August 2014, 01:20 (PST)	4th exfoliation event	D. Wyckoff personal communication (22 Sept. 2014)
22 August 2014	Crackmeter instrumentation (CM1, CM2, CMC) installed	
4 September 2014, ~02:00 (PST)	5th exfoliation event	D. Wyckoff personal communication (22 Sept. 2014)
2 October 2014	Crackmeter instrumentation (CM1, CM2, CMC) removed	
4 October 2014	Acoustic emissions monitoring (AE1-AE6) installed	
14–31 October 2014	Deep (~6 m) geotechnical drilling program	Condor Earth Exploration Report, 12 Dec. 2014 [64]
17–18 November 2014	Shallow (~2 m) geotechnical drilling program	Condor Earth Exploration Report, 12 Dec. 2014[64]
17 November 2014	Extensometer (EX1) installed	
25 March 2015	Acoustic emissions monitoring (AE1-AE6) removed	
1 April 2015	Dam repair begins	
16 April 2015	Begin reservoir filling	Twain Harte Lake Association presentation, 18 July 2015 [65]
30 April 2015	Dam repairs completed	
May 2015	Removal of loose exfoliation slabs at top of dome	
8 June 2015	Reservoir filled	Twain Harte Lake Association presentation, 18 July 2015 [65]
2–8 August 2015	6th exfoliation event—new cracking on rock and adjacent concrete	D. Wyckoff personal communication (7 Aug. 2015)
March-April 2016	Geotechnical rockbolt installation on north side of dome	
24 May 2016	Instrumented rockbolts for uplift forces (RB1, RB2, RBC) installed.	
7 June 2016, 12:50 PST	7th exfoliation event	S. Chung personal communication (8 June 2016)
22 July 2016, 18:25 PST	8th exfoliation event	T. Gillespie personal communication (22 July 2016)

^a^ The Rock Cracks, http://youtu.be/zS_ffU0v2QA

^b^ The Rock Cracks Again, http://youtu.be/Oo6bAqgYa9g

^c^ Real-Time Granite Exfoliation—The Rock at Twain Harte Lake, https://www.youtube.com/playlist?list=PLQjk-QqHkYh3Wb0WM2KtjikuBR0OEonL7

^d^ See Supplementary Movie 1

Additional fracturing occurred during the following summer (2015), but without the telltale explosive exfoliation previously observed. Then, in June and July 2016, two additional energetic exfoliation events occurred, buckling sheets up to 10 cm thick (Supplementary Fig. 1). Our rockbolt and extensometer equipment (Fig. 3), which had been installed in May 2016, captured the stress and deformation response. New exfoliation between 2014 and 2016 extended over a total area of 3030 m^2^, displacing 2340 m^3^ of rock (Fig. 2).

Our investigation at Twain Harte revealed no obvious trigger for the initial exfoliation events. We investigated several plausible mechanisms (e.g., seismic activity, drought, anthropogenic forcing; see Methods), but given that exfoliation occurred only during the hottest times of the year, in the absence of precipitation or other similar environmental forcing, we focused our efforts on the possibility of thermal triggering^18,35–37^, whereby stresses caused by thermal expansion of rock act to sub-critically, and then critically, fracture previously intact rock^20,21^.

### Spatial patterns of dome exfoliation

Exfoliation sheets generally increase in thickness and decrease in spacing at depth^5,11^, and Twain Harte Dome generally follows this pattern. On the northwest side of the dome, a single exfoliation sheet is located at 4 m depth and shallows towards the top of the dome, bifurcating to form two sheets at <1.5 m depth. Elsewhere, the main exfoliation sheet fracture surface parallels the topographic surface, with one to three thinner sheets comprising a total thickness of 1 m at interior sections (Supplementary Fig. 2) and only 10 cm at distal edges. The majority of early (2014) fracturing began in the northwest and moved first towards both the southeast and northeast, and then only to the northeast in 2015 and 2016 (Fig. 2). Exfoliation in the northwest was mostly confined to the single deep (4 m) fracture during the first (3 August 2014) event with only a few perpendicular fractures daylighting. As exfoliation subsequently progressed to comprise thinner, shallower sheets near the top of the dome, breakage occurred on multiple surface-subparallel fractures. Thus, the initial deep exfoliation on 3 August 2014 may have brought stresses closer to critical in the thinner, surficial sheets at the top of the dome.

Observations under the ruptured exfoliation sheets revealed a mix of weathered surfaces (some covered by several millimeters of soil; Supplementary Fig. 1) and fresh rock (Supplementary Fig. 2). Fracturing thus occurred through rupturing of rock bridges (fresh rock) between already formed macrocracks (weathered rock). We did not observe fractographic structures indicating fracture origin (e.g., mirror plane, plumose axes^38^) within freshly exposed surfaces. However, we did identify shingle-like structures (Supplementary Fig. 2; similar to those investigated elsewhere^7^) that suggest a roughly arcuate north-northwest to south-southeast fracture path, which correlates with the overall direction of the exfoliation events in 2014. Mapping of weathered remnants of older exfoliation sheets on Twain Harte Dome revealed two prior generations of exfoliation (S3, S4—Fig. 2). These sheets are comparable in scale (Fig. 2, inset) to those produced by the 2014–2016 events, and indicate differing relative exposure ages^39^. They therefore provide evidence that the events observed in 2014–2016 were characteristic of a process that repeats over longer time scales.

### Temporal patterns of dome exfoliation

Precursor audible cracking and centimeter-scale fracturing occurred prior to eventual uplift for several of the observed exfoliation events. Such observations are well-documented in quarrying and underground mining literature^25,40^, but can be generally tied to induced stress changes by mining activities themselves. Given that no such sudden stress changes occurred at Twain Harte Dome, these precursor signals are notable for providing some indication of imminent fracturing. During the latter half of the 6 September 2014 event and the two events in 2016 where sheet thicknesses were relatively thin (~10 cm), audible cracking occurred for several minutes prior to explosive exfoliation, allowing people to evacuate the dome. This sequence indicates a switch from subcritical to critical fracture propagation within the dome.

**Fig. 4:**
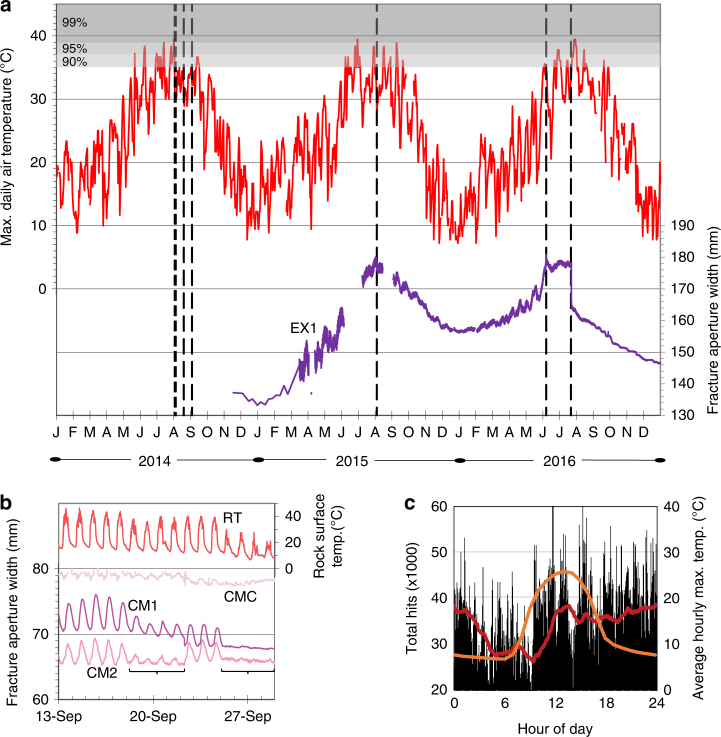
Time scales of critical and subcritical exfoliation fracture propagation. **a** Sheet deformation (EX1) correlates with maximum daily temperature with shaded quantiles indicating that all fracturing events (vertical dashed lines) occurred within 10-day time periods containing the hottest 10% of all days during the past 110 years. Initial fracture in 2014 occurred within 24 h of temperatures reaching the 99th percentile of hottest days on record. **b** Following cyclic diurnal fracture growth (CM1 and CM2), one exfoliation sheet collapsed (brackets) in 2014. Control signal (CMC) indicates small overall error (1 mm) compared to the sheet signals. **c** Total acoustic emission hits (*n* = 48.7 × 10^6^) from six sensors (Fig. 1c) over 6 months (October 2014–March 2015) indicate subcritical cracking was ongoing following the 2014 events; sharp increase in 3-h hit running average (red line) peaks with maximum temperatures (orange line)

In all cases, fracturing occurred only during the summer (June through September) (Fig. 4a) and were preceded by 10-day periods with some of the highest temperatures on record near the site. In 2014, initial fracture occurred within 24 h of air temperatures reaching the 99th percentile of highest temperatures recorded (Fig. 4a). Rock surface temperatures were even higher (>50 °C) during these time periods.

On a daily cycle, deformation of the uppermost sheets provides direct evidence for unambiguous subcritical crack growth that closely tracks diurnal temperature. For example, following the first four exfoliation events in 2014, one exfoliation sheet continued to undergo ~5 mm daily deformation (as measured by crackmeters), with opening and closing of sheet fractures coincident with strongly fluctuating (*ΔT* = 25 °C) daily temperatures (Fig. 4b). This pattern continued through the fifth exfoliation event (on 4 September 2014) until initial (18 September 2014) and then irreversible (25 September 2014) collapse occurred during a period of 20 °C overall cooling. Total sheet collapse resulted in 5 mm of permanent deformation, crushing the crackmeter instrumentation (which could only be extricated by physical removal of the overlying rock). The sequence of cyclic deformation resulting in permanent settlement of the sheet in the absence of an explosive event indicates the prevalence of subcritical crack growth during this time. This process also occurred during the summer of 2015 when visible daily crack propagation occurred on the north side of the dome, but again in the absence of an explosive exfoliation event.

Periods of subcritical fracturing are also indicated by AE data (Fig. 4c). These data record tens of thousands of AE hits that occurred on the dome (with hits taken as an indication of rock damage^17^), even during a period of exfoliation quiescence that followed the energetic and slow collapse events earlier in 2014. As such, this AE activity may have been a precursor to the visible fracturing observed later in 2015. At the hourly scale, AE signals show greatest evidence for microfracture in the afternoon and evening (Fig. 4c). Insolation-related subcritical fracture may occur at any time of day depending on the geometry and aspect of individual sensors, and environmental conditions, but is generally most efficient and strongest during periods of rapid warming (mid-day during the winter period when AE monitoring occurred) and cooling (late evening)^41^.

### Stress and deformation precursors

**Fig. 5:**
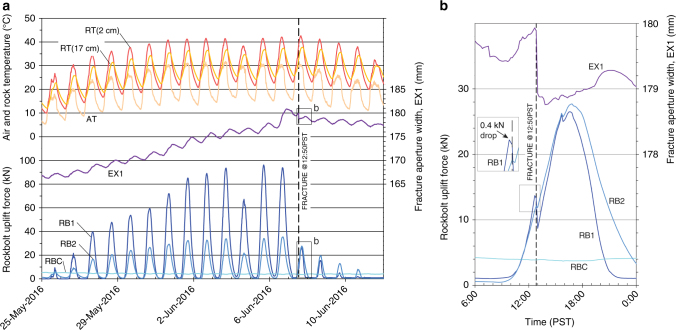
Uplift signals and temperature response of uppermost rock sheet prior to the 7 June 2016 exfoliation event. **a** Rising air temperatures results in amplified rock temperatures, with slight (1–2 h) delays in temperature change and consequent increases in rock uplift forces and sheet deformation. When maximum temperatures level out (early June), uplift forces and deformation continue to increase until energetic fracture (dashed line). **b** Fracture was accompanied by a 1 mm instantaneous settlement of the uppermost sheet and force drop of 5 kN. Instrumentation subsequently continues to measure stresses from other parts of the dome. A possible precursor uplift force drop (indicative of an overall stress drop) was captured in the 10-min prior to energetic fracture (inset box). Instrument error (2*σ* = 0.8 kN) as determined from RBC is far below the uplift force magnitudes reached during this time. RB# rockbolt uplift force, RBC rockbolt uplift force control, EX1 extensometer deformation, AT air temperature, RT rock temperature (depth indicated); instrument locations as shown in Fig. 1

Stress and deformation (i.e., sheet fracture aperture opening and closing) monitoring data captured during two of the events indicate a direct link between exfoliation and both high and cumulative temperature increases. During the 7 June 2016 event, rockbolts (RB#) and extensometers (EX1) installed across the main exfoliation sheet (Fig. 3) measured diurnal and cumulative increases in uplift force (i.e., with associated uplift tensile stresses) and resultant deformation (Fig. 5). Over a 2-week period, uplift forces increased from a few to 95 kN (RB1-Fig. 1c) where open fractures are shallow (at 0.7 and 0.9 m depth; Fig. 3b) at a point near (15 m) to the eventual location of fracture. Further (25 m) to the west (RB2; Fig. 1c), where open fractures are deeper (1.1 and 1.5 m depth; Fig. 3b), uplift forces reached roughly a third of that magnitude (33 kN). During this time, daily maximum air temperatures increased by 18 °C, with rock surface temperatures reaching 42 °C.

Overall sheet deformation on the north side of the dome (EX1) increased concomitantly with the rockbolt signals, with 14 mm of cumulative uplift (Fig. 5a). These signals closely tracked air and rock temperatures, although, notably, uplift forces and sheet deformation continued increasing even during a three-day period (1 June 2016 – 3 June 2016) in which maximum temperatures leveled off, suggesting that the rock dome cumulatively stores thermal energy. Energetic fracture occurred at 12:50 PST (13:50 local time), just prior to temperatures peaking after a steady 2-week increase. A 5 kN force drop and 1 mm of sheet settlement (as measured close to the exfoliation area; Fig. 5b-RB1) ensued. The temporal resolution of our data (10 min) does not allow us to determine if stress or deformation precursors were exhibited prior to the force drop. However, a small (0.4 kN) force drop was recorded at RB1 during the 10-min period prior to fracture (see inset in Fig. 5b), suggesting that the presumed spontaneous exfoliation may actually have been portended by precursor signals.

Following the energetic exfoliation and minor settlement of the sheet, uplift forces and sheet fracture aperture decreased, mirroring the temperature response. An exception is the proximal (RB1) force response to the exfoliated area, where uplift forces first decreased to match the distal uplift force response at RB2 on the day of fracture, and then continued to decrease, eventually reaching a baseline (zero force) condition on 11 June 2016 (Fig. 5a). Notably, positive measured uplift forces resumed as temperatures rose in late June 2016, eventually reaching values roughly a third of those previously recorded and resulting in the subsequent adjacent exfoliation (22 July 2016; Fig. 2), with 12 mm of measured sheet settlement at EX1. Thus, the two 2016 events are likely related to the releasing of thermal stresses accumulated during the summer.

### Thermal forcing of subcritical cracking and critical fracture

To understand exfoliation mechanics, we reconstruct the tectonic and thermally induced fracture conditions of exfoliation domes using subcritical fracture research^20^ and work performed on similarly detached exfoliation sheets in nearby Yosemite National Park^13,21,42^. First, it is useful to verify if thermal cycling via heat transfer is capable of heating exfoliation sheets at depth. Although we lack rock temperature data at the full depth of presumed fracture (at least 1 m and potentially up to 4 m), our in situ rock temperature measurements (Fig. 5) show the thermal evolution at shallow depths, with diurnal temperature changes of 20 °C at 2 cm depth and 12 °C at 17 cm depth. Further, total temperature changes (i.e., maximum minus minimum temperature) over 14-day time periods caused the exfoliation sheets at Twain Harte Dome to heat up much more substantially, with increases of up to 30 °C and 25 °C at 2 cm and 17 cm depth, respectively. These depths capture the thermal signature of many of the explosive exfoliation events; uplifted surficial sheet thicknesses were on the order of several to 30 cm during both 2014 and 2016. Thus, our data show that exfoliation domes undergo heating through sheet thicknesses observed to result in fracture.

The direct result of heating rock is expansion, a process well-studied and quantified^18,21,41,43^. Research^20,44^ shows that cumulative fracture growth can occur under typical present-day meteorological conditions in many settings. The only constraint is that the thermally generated stress intensity factor (*K*_I_, the mode I, or tensile, rock fracture stress intensity factor) is above the subcritical cracking threshold (*K*_Ith_), which is estimated^17^ to be 10–20% of the fracture toughness, *K*_IC_—a measurable rock-intrinsic property^45^. We can determine if stress values reached *K*_Ith_ using the stress intensity approach from fracture mechanics theory (see Methods) by calculating the thermally generated tensile stresses and resulting *K*_I_ acting on the exfoliation sheets. Predicted tensile stresses from thermal expansion acting at the fracture boundary have been estimated to be 100 to 300 kPa for grain-scale geometries^44^ and lower (on the order of only several kPa) for cliff and dome-scale fractures^21^. Our rockbolt monitoring data allow us to explicitly calculate these stresses (16 and 55 kPa—see Methods) from the measured force data for one event (7 June 2016) at Twain Harte Dome and likewise to compute a resulting *K*_I_ between 0.05 and 0.18 MPa√m.

How does *K*_I_ compare to *K*_Ith_ and *K*_IC_? We do not have site specific values for *K*_IC_ at Twain Harte, but laboratory values^21^ (0.7 MPa√m) from similar nearby granodiorite rocks indicate that the thermally generated stress intensity factor at Twain Harte straddles the range of *K*_Ith_ (10–20% *K*_IC_* = *0.07–0.14 MPa√m). In addition, the *K*_Ith_ = 10–20% *K*_IC_ estimate may represent a maximum due to experimental time scales, and could be lower over geologic time^46^. Thus, non-explosive exfoliation will be an ongoing (subcritical) process from thermally induced stresses—a process potentially occurring in rocks elsewhere^10^. However, subcritical crack growth does not explain the explosive (critical) nature of many of the events at Twain Harte, namely, how did *K*_I_ reach *K*_IC_? Here, we must assume that regional tectonic stresses exist within the rock (i.e., similar to those measured in a nearby, and geologically and tectonically analogous, part of the Sierra Nevada mountain range^47^) and that thermally generated stresses acted in superposition^45^ to these. In situ tectonic stresses have been measured in many granitic exfoliation sheet settings across the United States, and the average values (12.2 MPa and 16.5 MPa) resulting from these studies^12,47^ bracket the value (13.9 MPa) reported from a shallow (0.6 m) depth in the Sierra Nevada^47^. Using this regional measurement results in a tectonic stress intensity factor (*K*_I(tect)_) equal to 0.62 MPa√m (see Methods). Thus, the rock at Twain Harte could have easily reached *K*_IC_ (0.7 MPa√m), when thermal stresses intensities (*K*_I(therm)_ = 0.05 and 0.18 MPa√m) also acted on the dome. In many ways, this superposition mechanism, where thermal stresses act in concert with an existing stress regime, is similar to that hypothesized for tidal-triggered earthquakes^48,49^, where seemingly small stresses can cause ultimate fracture. Thus, we view the events at Twain Harte as examples of an initially tectonically stressed rock undergoing a transition from first long-term thermally driven subcritical processes, to that of critical fracture at times of maximum temperature.

## Discussion

Can fracture of exfoliation domes such as witnessed at Twain Harte Dome be predicted? Research on rockburst phenomena in deep underground mines and tunnels indicates that understanding precursors for energetic fracture remains challenging. The use of microseismic and acoustic emissions monitoring^26^ and in situ stress measurements^27^ shows promise for updating existing empirical models of prediction^50^, however these techniques may not resolve the much smaller stress changes that occur from thermal cycles under no significant overburden stress. It is possible that the stress and fracture conditions measured for the events at Twain Harte Dome could be applied to other surficial exfoliation events; our monitoring indicates that stress measurements provide a more meaningful determination of expected exfoliation than deformation measurements. However, the interplay between subcritical and critical fracture is paramount. The 2015 events at Twain Harte Dome had no explosive fracture and only underwent subcritical crack growth, most likely due to stress readjustment following the 2014 exfoliations; these events set the stage for critical explosive fracture in 2016. On the other hand, understanding why these events began specifically in 2014 is limited to the observation that the first event occurred within 24 h of the hottest temperatures on record and that—all other things being equal—all stresses occurring above the subcritical cracking threshold will eventually lead to critical failure as cracks increase in length^20^. Although others^1–3,5,7–9,23,29,30^ could only speculate on the origin of these stresses, we have shown that fracturing and energetic exfoliation can be expected during the hottest periods of the year, and that subcritical fracturing—likely over long time scales—is an important contributing factor. Our study therefore validates that the insolation hypothesis for exfoliation sheet fracture is not only a viable mechanism, but also a contemporary process in the evolution of this and potentially other rock domes.

## Methods

### Study area geology and dam history

Twain Harte Dome is one of several granitic domes^51^ located on the west slope of the Sierra Nevada Mountains of California (Fig. 1). The rock comprising Twain Harte Dome is a Mesozoic-age medium-grained granodiorite with an average grain size of ~1–2 mm (Supplementary Fig. 3). The rock dome forms the left (south) abutment of a 11-m-tall, 100-m-long, concrete multiple-arch dam that impounds 11 hectare-meters of water. The dam was constructed in 1928; inspection of pre-construction site photographs indicates the dome geometry did not significantly change as a result of construction except where the dam is keyed into the dome. The reservoir (i.e., Twain Harte Lake) is used for recreation during the summer months and pool level is generally kept full year-round—inflows from winter precipitation and spring snowmelt spill along the length of the dam crest to the west-draining creek on the north side of the dome. No anomalous activities related to the dam were noted by the lake manager (D. Wyckoff, 2014, personal communication) or local residents in the years, months, and days leading up to the beginning of recent exfoliation in 2014.

### Direct visual observations

Nearly all of the major exfoliation events described herein (Table 1) were directly observed by visitors to the lake (reservoir) or employees working for the Twain Harte Lake Association. For all events, we conducted interviews with eyewitnesses, reviewed video resources, and made post-event reconnaissance visits to the site to document fracturing. For events 1, 2, and 3 (as listed in Table 1), video was captured by bystanders on the dome, as well as from a fixed camera at a neighboring lake cabin located ~140 m from the dome. Event 2 was observed and videoed by coauthor S.W.L. For events 4 and 5, reports of sudden, audible cracking were made by a security guard working at the site. Event 6 was documented by daily observation of the dome by the Twain Harte lake manager (D. Wyckoff, 2015, personal communication), and events 7 and 8 were widely observed by lifeguards and recreationists at the dome (see references in Table 1). These observations provide details on the timing, location, fracture pattern, and audible cracking sounds for each of the events.

### Dismissal of possible seismic and anthropogenic triggers

Given the exclusively summer timing of exfoliation events at Twain Harte Dome, we purposefully aimed our studies at investigating triggers that coincided with seasonality influences. Admittedly, there are many possible triggering mechanics for exfoliation, but most appeared to be non-influential to the observed events (e.g., no events coincided with rainfall or seepage-related processes, and examination of on-site humidity data—with measurements beginning in May 2016—revealed that the two exfoliations in 2016 occurred each at both high (~65% on 7 June 2016) and low (~20% on 22 July 2016) periods of relative humidity. Thus, we focused on those triggering mechanisms that might be most plausible during the times when exfoliation events occurred.

We investigated the possibility that nearby seismic activity could contribute to the Twain Harte exfoliation events by searching (http://earthquake.usgs.gov/earthquakes/search/, accessed 1 May 2017) for precursor seismic signals. This search revealed a total of thirteen seismic events occurring within a 50 km radius in the 30 days preceding the exfoliation events of each season (eight in 2014, four in 2015, and one in 2016). Of these, all but three were associated with blasts at nearby (16–30 km) quarries. We noted that blast signals occur in all months of the year and in years when no exfoliation occurred, and accordingly considered them unrelated to the events at Twain Harte. The three other signals were shallow (6 to 27 km depth), small magnitude (M1.8 to M2.4) earthquakes located between 14 and 36 km from Twain Harte Dome. Due to the small magnitude and lack of direct temporal coincidence (all earthquakes occurred more than 10 days prior to an exfoliation event), we judged these to be unrelated to the events at Twain Harte. Low-level seismicity is typical for the region with small magnitude (<M3.0) earthquakes occurring during all months of the year (62 earthquakes with magnitude ranging from M0.9 to M2.8 were recorded in a 50 km radius of the site during the ten years prior to the first exfoliation event).

To identify possible anthropogenic triggers, we talked with site personnel and long-time visitors to the lake. Interviews with the lake manager (D. Wyckoff, 2014, personal communication) indicated no dam-related precedent for the events; the dam was constructed in 1928 and the reservoir had undergone filling and draining cycles without any visible fracturing noted on the dome. It is possible that annual filling and lowering cycles of the reservoir level, or simply the loading of the dam and reservoir themselves, could have caused some stress increase in the dome. However, in the years prior to the 2014 exfoliation events, the reservoir was generally kept full to near-full for most of the year (precluding cyclic reservoir loading). Further, the additional exfoliation events in 2016 occurred following dam repairs (a series of vertical boreholes were drilled between the dam and the new areas of exfoliation sheets) that essentially decoupled the dam from the upper surface of the rock (precluding direct dam loading). This leaves static reservoir loading as a long-term, time-dependent possibility for stress increases to the dome, which cannot be entirely ruled out. However, this also appears to be only a speculative possibility given that any resultant water loads cannot act directly on the surface of the dome (as heat can); the vast majority of ruptured exfoliation sheets were well above the maximum reservoir level.

No abnormal activities had occurred at Twain Harte Dome prior to the beginning of exfoliation events, with the exception that ~300 people had been present on the dome just a day before the first exfoliation event for an annual community gathering. However, this type of loading (estimated to be ~0.2 kPa based on 300 people, each weighing 80 kg and distributed over roughly 25% of the top area of the dome) had also occurred during previous years without any noted rock fracture of the dome. Further, no similar episodes of large numbers of people on the dome occurred prior to any of the other exfoliation events. Thus, we conclude that this loading condition does not explain the origins of exfoliation.

Prolong drought affected the western United States from 2012 to 2016 (https://www.drought.gov/drought/west, accessed 17 May 2017), with low cumulative precipitation, record thin snowpack in the mountains, reduced reservoir levels, and increased groundwater pumping. The loss of water mass in the region drove vertical land surface displacements (as recorded by global positioning systems) upwards by between a few to a maximum of 15 mm in the central Sierra Nevada (where Twain Harte Dome is located) as a result of elastic crustal deformation^52^. It is possible that uplift of the Sierra Nevada could have changed the state of regional, and likewise local, tectonic stress, thereby altering the stress field at Twain Harte Dome. However, although the timing of drought-induced uplift broadly coincides with exfoliation at Twain Harte Dome, it does not explain the strong seasonality observed there.

In addition to regional groundwater pumping, we found that localized pumping from a nearby 140-m-deep well occurred in the months prior to several of the exfoliations. To determine if this may have played a role in exfoliation at Twain Harte Dome, we reconstructed monthly groundwater extraction volumes by back-calculating water usage from electricity-provider billing statements (with energy usage, in kW) for the time period 2009 through 2016, combined with power demand and efficiency calculations for the groundwater well pump (Berkeley 6T-200, 15.2 cm diameter pump operating at 67% efficiency at 0.51 m^3^ min^−1^ with 90.21 m water head). These calculations showed that ~74,000 m^3^, 127,000 m^3^, and 16,000 m^3^ of water were extracted in the months leading up to the 2014, 2015, and 2016 exfoliation events. Water was withdrawn from 140 m depth below and 370 m to the east of the dome, and was used to fill the lake; thus, the only actual losses to the local fractured rock mass were from evaporative losses from the lake. Similar quantities of water (50,000 m^3^) had been withdrawn in previous years without incident (e.g., 2012 and likely several times prior to 2009 when electricity records are not available) and there is not a direct temporal correlation between groundwater withdrawal volume and exfoliation (i.e., the largest water volume was extracted in 2015 when no energetic exfoliation occurred). Furthermore, we could not identify a physical mechanism by which local groundwater lowering more than 100 m below a rock dome would cause a significant stress increase at the dome surface. Although ground subsidence and changes in surface stress can occur from groundwater pumping, this process is more often regulated to aquifers composed of loose sediments (rather than hard rock such as found at Twain Harte). Although it cannot be entirely ruled out, we do not expect that opening of deep underground joints or depressurizing of these joints would have a significant effect on the surficial bedrock more than 100 m away.

These conclusions, coupled with the realization that similar exfoliation events have occurred in the Sierra Nevada^2,9,42^ where no groundwater extraction has occurred (e.g., in wilderness areas of Yosemite National Park), led us to dismiss groundwater extraction as a primary causative factor for the events at Twain Harte. More likely is that groundwater extraction occurred concomitant with lake filling during the hottest summer months.

### Topographic and fracture mapping

We used terrestrial lidar and differential global positioning system (GPS) surveying to construct a high-resolution topographic model of the dome and to map visible surface fractures from the exfoliation events. Our lidar data set, collected using a Riegl Z420i laser scanner from 14 scan positions surrounding and on top of the dome, consists of 25 × 10^6^ points of the dome surface. We collected fracture location data of the first four events using a pair of Topcon Hiper + dual-frequency RTK receivers—points on fractures were collected at ~1.5 m spacing along the visible aperture width (>~ 0.5 mm) of surface cracks. We updated this mapping using field observations made following the other exfoliation events and supplemented these observations with those provided to us by others (notably scale maps drawn by personnel from the California Division of Safety of Dams). During a field visit on 8 September 2014, we also measured the aperture, surface perpendicular depth, orientation (strike and dip), and sheet thickness (where measurable) along visible fractures. For base map generation, we collected high-resolution (1.65 cm per pixel), aerial imagery in March 2017 using a 3DR Solo unmanned aircraft system (UAS) with a Canon Powershot S100 (12.1 megapixels) attached in nadir position (i.e., pointing straight down). We processed this data using Agisoft Photoscan software (v.1.2.6) and achieved a 10 cm RMSE with 21 georeferenced ground control points.

### Exfoliation sheet mapping

To document the scale(s), extent and relative age of exfoliation sheeting across the dome surface, we mapped the surface exposure of all stratigraphically distinct remnants (S3 & S4) of exfoliation sheets overlying the exfoliated sheet(s) detached in the 2014–2016 events (S2). The lowest sheet whose top surface was exposed by natural breakup and subsequent removal from the site by construction activities of S2 was termed S1. Measurements of S1 thickness cannot be made because they have yet to either form or be exposed. Sheet thickness (see Fig. 2, inset) was measured at several (S2: *n* = 16, S3: *n* = 10, S4: *n* = 9) representative locations and general observations were made of weathering characteristics (surface relief, dissection, micro-scale cracking) of each of the mapped exfoliation sheets^39^.

### Exploratory drilling

As part of the repairs made to the dam and infrastructure located on the dome, 59 geotechnical boreholes were drilled into the dome. The boreholes, installed using 5.1 cm diameter percussion drilling techniques, revealed the depth to fracture, and thus the resultant geometry of the main arcuate detachment sheet on the north side of the dome. These measurements were supplemented with visual observations of the thinner (<30 cm) parts of the exfoliation sheet that could be directly observed and measured where exposed in the many slabs of rock that had been lifted at the surface.

### Acoustic monitoring

We installed six acoustic emission (AE) sensors (Physical Acoustics Corporation PK15I; AE1-6—Fig. 1c) on the dome using established methodologies^53^. Monitoring occurred from 4 October 2014 to 24 March 2015, during which time the dam underwent repairs. The AE instrumentation was removed following construction activities and reopening of the dome to lake visitors. During the monitoring period, when the elastic wave measurement received from a single AE sensor exceeded a pre-defined threshold value (45 dB as suggested by the AE manufacturer for this application), data were recorded and referred to as an acoustic emission hit. Although AE monitoring of rock cracking under natural, non-loaded conditions is not common, it is increasingly undertaken with good results^41,54–58^. Further, it is well-accepted from rock physics applications^17,59^ that hit rate is proportional to the damage incurred by subcritical cracking in rock. Here, the timing of each AE was processed using Physical Acoustics Corporation AEwin software; examination of other features of the AE signal (e.g., energy or amplitude) was beyond the scope of this study.

### Deformation and environmental monitoring

To capture post-event deformation, we deployed two vibrating wire strain gage crackmeters (Geokon 4420-1×-50; CM1, CM2—Figs. 1c and 3c, Supplementary Fig. 4) in prominent fractures on the dome surface on 22 August 2014, 48 h after the fourth event. Depth (i.e., exfoliation sheet thickness) at CM1 and CM2 were ~7 and 26 cm (Fig. 3b), respectively, with fracture aperture widths of ~65 to 75 mm (Fig. 4b). An identical control device (CMC) was installed in a fixed position near CM2, but not in direct contact with the deforming exfoliation sheets. The gages measured expansion and contraction of the uppermost fractured exfoliation sheet to an accuracy of 0.05 mm and have been used in previous exfoliation studies^21^. These instruments collected data at 5 min intervals for a period of 42 days until they were crushed by detachment and collapse of the sheet. Raw sheet deformation signals (Fig. 4b) were well above the error level identified with the control device (1 mm) from thermal expansion of the device and vibrating wire sensor themselves. Crackmeters did not capture any signs of the fifth exfoliation event on 4 September 2014, presumably because of the event’s small size (D. Wyckoff, 2014, personal communication).

As part of the remediation of the dam, a single 5.6-m-deep, 3-channel extensometer (Geokon 4450 with accuracy of ±0.02 mm) was installed on 17 November 2014 (EX1—Fig. 1c, Fig. 3d, Supplementary Fig. 5). Depth of individual channel installations were 0–1.7 m (Channel 1-1), 0–3.2 m (Channel 1–2) and 0–5.6 m (Channel 1–3). All three channels of the extensometer span two open fractures at 0.58–0.64 m and 0.69–0.76 m borehole depth (Fig. 3b) for a total combined fracture opening (6 cm + 7 cm) of 13 cm. However, the fractures soon grew larger, reaching a maximum of ~18 cm during the summer of 2015 (Fig. 4a). The extensometer was initially installed to periodically (at approximate 10 day intervals) measure potential deformation between the surface and three depths (1.7 m, 3.2 m, and 5.6 m) during the repair of the dam. We installed a data logger on the instrument on 17 March 2015 to collect high temporal resolution (5-min) data following the repairs. The sampling rate was then changed to 10-min on 24 May 2016. The signals between channels are nearly identical such that we only plot the uppermost channel (Channel 1-1) installed at 1.7 m depth. Data for all three channels are presented in Supplementary Data 1, which provides both raw (calibrated) and final (calibrated and temperature-corrected) signals using standard manufacturer protocols for temperature correction.

We installed vibrating wire strain gage instrumented rockbolts (Geokon 4910; RB1, RB2, RBC—Figs. 1c and 3e, Supplementary Fig. 6) on 24 May 2016, 14 days before the first energetic exfoliation event in 2016. Boreholes were drilled with a 5.1-cm-diameter percussion bit to a depth of 152, 208, and 71 cm for RB1, RB2, and RBC, respectively. Grade 75 steel, all thread, #8 (2.54-inch diameter) rebar with a vibrating wire strain gage mounted longitudinally at the bar center were grouted into the bottom of the holes over a 56 cm length for RB1 and RB2, and 15 cm for RBC. The signal rockbolt bars spanned two open fractures at 0.71–0.74 m and 0.81–0.91 m for RB1 and 1.14–1.19 m and 1.42–1.50 m for RB2 (Fig. 3b); the control rockbolt (RBC) did not penetrate the exfoliation sheet fractures. A mounting plate (washer) and torqued nut finished the placement at the surface with an initial preload of 5 to 6 kN following the installation. The preload from rockbolt tightening is small compared to the forces generated by rock uplift, and regardless, are indicative of measureable magnitudes of rock stress (any forces measured above the rockbolt tightening preload indicate the likely presence of stresses below this point as well). In all cases, the force change difference from installation is reported and is therefore a minimum uplift force compared to background conditions, which can only be ascertained when thermal sheet deformation is maximally inward during the winter months (December–March). Tightening of the bolts was required several times over the 2016 summer following the exfoliation events to account for loosening caused by exfoliation event deformations (i.e., sheet settlement). This did not affect the results for the same reasons as presented for the preload forces. Although the vibrating wire strain gages on the rockbolts measured deformation, our instruments were factory calibrated to the stiffness and diameter of the steel rebar and thus provided force measurements. Forces are converted to uplift tensile stresses through analysis of exfoliation sheet geometry (see Methods—“Fracture stress intensity analysis” section). Errors in force readings from thermal deformation of the rebar are minimal (2 kN) and therefore not applied to the presented data signals (Fig. 5). The control device was preloaded to the same preload force (5 kN) as for the measurement devices (e.g., RB1 and RB2), with data indicating that instrumental error (2*σ* = 0.8 kN) was far below the force magnitudes reached during the monitoring period.

We originally (from August 2014 to May 2016) measured near-rock-surface air temperature (as a proxy for surface rock temperature) using pendant-type data loggers (Onset Hobo UA-002-64) installed at five locations (north, east, south, west, and top) on the dome. Owing to construction activities related to dam repair, a full record is incomplete for any one sensor, but variability was small between sensors due to their close proximity and similar aspect. We thus present data only from the North sensor (RT, Fig. 1c) because it was closest to the majority of exfoliation events. In May 2016, we installed a solar-radiation-shielded air temperature and humidity sensor (Vaisala HMP60; AT/RH—Fig. 1a) and two drilled and epoxied rock temperature sensors (Campbell Scientific 107; RT—Fig. 1c) at 2 cm and 17 cm depth near the same location as the pendant logger on the north side of the dome. These instruments provide the data for Fig. 5 and for rock temperatures reported for the exfoliation events in 2016.

### Fracture stress intensity analysis

During the 7 June 2016 exfoliation event, rockbolt uplift forces across the partially attached, deforming sheet reached 33 kN and 95 kN at 25 m (RB2) and 15 m (RB1) distance from the nucleus of exfoliation (Fig. 2), and across minimum surficial sheet thicknesses of 1.14 and 0.71 m, respectively (Fig. 3b). We assume that these uplift forces are directly related to thermal forcing due to their synchronous relationship with diurnal temperature fluctuations. Because the measured uplift forces act on a part of the sheet that did not fully rupture, we compute tensile forces at the crack tips of the rupture site by two methods, each involving an extrapolation of data from RB1 and RB2. The first method of computation assumes that tensile forces increase linearly with decreasing distance to the site of rupture (i.e., 95 kN–33 kN force difference over a 10 m span between RB1 and RB2 = 6.2 kN m^−1^) thereby indicating that uplift forces are maximum at the site of fracture. The uplift force, *U* at the 7 June 2016 site is then 188 kN (95 kN at RB1 plus 6.2 kN m^−1^ increasing over 15 m between RB1 and the site of exfoliation gives *U* = 95 kN + 6.2 kN m^−1^ × 15 m = 188 kN). The second method of tensile stress computation assumes that the uplift forces are either linearly or exponentially related to the thickness of the slab (which thins toward the site of rupture). Assuming a linear relationship, and for the geometry at RB1 and RB2, we have uplift force *U* = −144.2*d* kN m^−1^ + 197.4 kN, and for a thickness *d* = 0.1 m, the uplift force is 183 kN. We can also fit an exponential function to the two data points from RB1 and RB2 (that is, RB1 load of 95 kN with 0.71 m sheet thickness and RB2 load of 33 kN with 1.14 m sheet thickness), which results in *U* = 544.4 e^−2.459*d*^, which again for *d* = 0.1 m, results in *U* = 426 kN. Thus, the estimated uplift force at the site of rupture using both of these methods is between 183 kN and 426 kN. Whereas we cannot know the magnitude of the uplift force at the crack tips of the rupture sheet with certainly, our measurements provide at least a guideline based on real data. Applying the computed force evenly across the geometry of the exfoliation sheet (7.1 m by 2.2 m; Supplementary Fig. 1), and noting from field observations that the sheet was only partially (50 to 75%) attached to the parent rock prior to fracture, the acting tensile stress change (Δ*σ*) is between 16 and 55 kPa (full range of all estimates of *U*), which acts to open the exfoliation fracture in a mode I (tensile) direction. For the 7.1 m long sheet, the half crack width (*a*) is 3.55 m prior to the 7 June 2016 exfoliation. Implementing the stress intensity approach from linear elastic fracture mechanics theory^45^ (*K*_I_* = *Δ*σ√*π*a* for an idealized two-dimensional isolated crack), the resulting thermally generated stress intensity factor, *K*_I(therm)_ is between 0.05 and 0.18 MPa√m.

To estimate how the dome reached a state of critical fracture, we assume that the dome as a whole is under a similar compressive tectonic stress regime as measured by overcoring technique in nearby (~70 km to the southeast) Tuolumne Meadows located in Yosemite National Park^47^. Because the two locations (Tuolumne Meadows, California, and Twain Harte Dome) are not decidedly different (i.e., the sites both exhibit shallow and topographically parallel exfoliation sheets, the lithology is composed of similar Mesozoic-age granitic plutons, there are no major intervening faults between the two locations, and this portion of the Sierra Nevada mountain range has had a similar tectonic history^60,61^ and is experiencing similar modern uplift rates^62^), the compressional stress magnitude measured at the Tuolumne Meadows site^47^ (13.9 MPa) may be taken as broadly applicable to the tectonic setting in the region of Twain Harte Dome. Taking these stress measurements as the compressive (negative) stress, *P* (−13.9 MPa), together with values of measured dome curvature, *κ* (−0.007 to −0.027 m^−1^) and gravitational components of stress from the sheet (rock mass density, *ρ* = 2670 kg m^−3^, gravity, *g* = 9.81 m s^−2^, slope, *β* = 28°, and a 1 m sheet thickness, *d*), we applied a curvature-based tensile stress formulation^12^ (*Ψ* = [*κP – ρg*cos*β*]*d*) to compute the resulting tensile stress (*Ψ*) at the crack tips. Using these values, we calculate maximum tensile stresses on the order of 0.35 MPa. Now, using the stress intensity approach presented previously (*K*_I_* = *Δ*σ√*π*a*) with an estimated original (3 August 2014) fracture half length, *a* of 1 m (a very poorly constrained but rough estimate on what the original fracture length may have been at the time of energetic exfoliation), we obtain a tectonically driven tensile fracture stress intensity factor, *K*_I(tect)_ of 0.62 MPa√m. We note that these calculations make a number of broad assumptions (namely an overly simplified analytical solution for the actually more complex fracture geometry, and a speculative estimate of the initial fracture length), but are still useful for showing how a combination of tectonic and thermal stresses can reach the critical fracture toughness.

### Temperature climatology

We investigated long-term temperature trends at Twain Harte Dome with climatology data from the Sonora, California station^63^ (GHCND Station ID# USC00048353) that records meteorological observations spanning 110 years (1906–2016). Sonora is ~14 km from Twain Harte Dome and 575 m lower in elevation. Climate in Sonora, as derived from data spanning a period from 1984–2013, is characterized by a mean annual temperature of 15 ± 7 °C, with average monthly highs and lows ranging from 34 ± 3 to −0.1 ± 3 °C, respectively. Average daily range of temperature is 17 ± 5 °C. A comparison of 2 years of our temperature data (daily averages) from Onset Hobo pendent-type data loggers recording near-surface rock temperature at Twain Harte Dome, with that from the Sonora station shows good agreement (*R*^2^ = 0.87; *p*-value < 0.001). We calculated quantiles for maximum daily temperatures over the 110-year period at the Sonora station (presented in Fig. 4a) using JMP software.

### Data availability

The data generated and analyzed during the current study are available in Supplementary Data 1 and/or from the corresponding author on reasonable request.

## Electronic supplementary material

Supplementary Information

Description of Additional Supplementary Files

Supplementary Movie 1

Supplementary Data 1
