# Supplementary material for: Thermal influences on spontaneous rock dome exfoliation
Source: Nat Commun. 2018 Feb 22;9:762. doi: 10.1038/s41467-017-02728-1 (PMC5823905; doi:10.1038/s41467-017-02728-1)
Supplement: Supplementary file 1 — Supplementary Information [file 41467_2017_2728_MOESM1_ESM.pdf]

Supplementary Figure 1

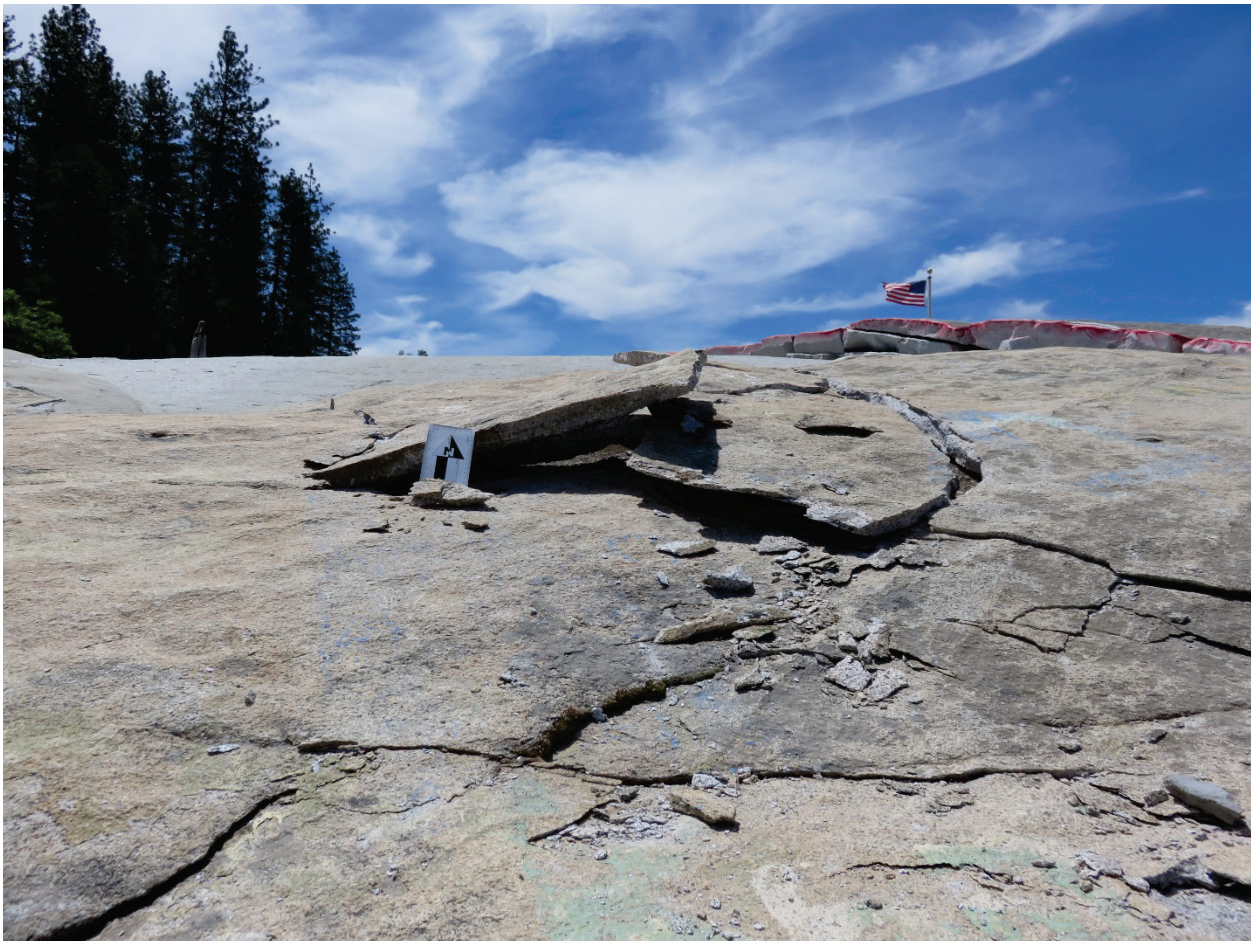

Supplementary Figure 1. Tent structure formed by energetic exfoliation fracture on 7 June 2016 at Twain Harte Dome. Total fracture occurred over a 15.6 m^2 area with 25 cm uplift of a 10 cm thick granodiorite exfoliation sheet. The event thrust rock fragments and dust 2 m in the air and was preceded by several minutes of audible cracking sounds. Scale bar is 15 cm. Several-millimeter-thick deposit of dark brown soil is visible to the right of the scale bar and under the tilted rock sheet indicating that the sheet was partially detached for some time prior to rupture. Exposed exfoliation sheets from energetic events in 2014 are visible in the background beneath the flag.

Supplementary Figure 2

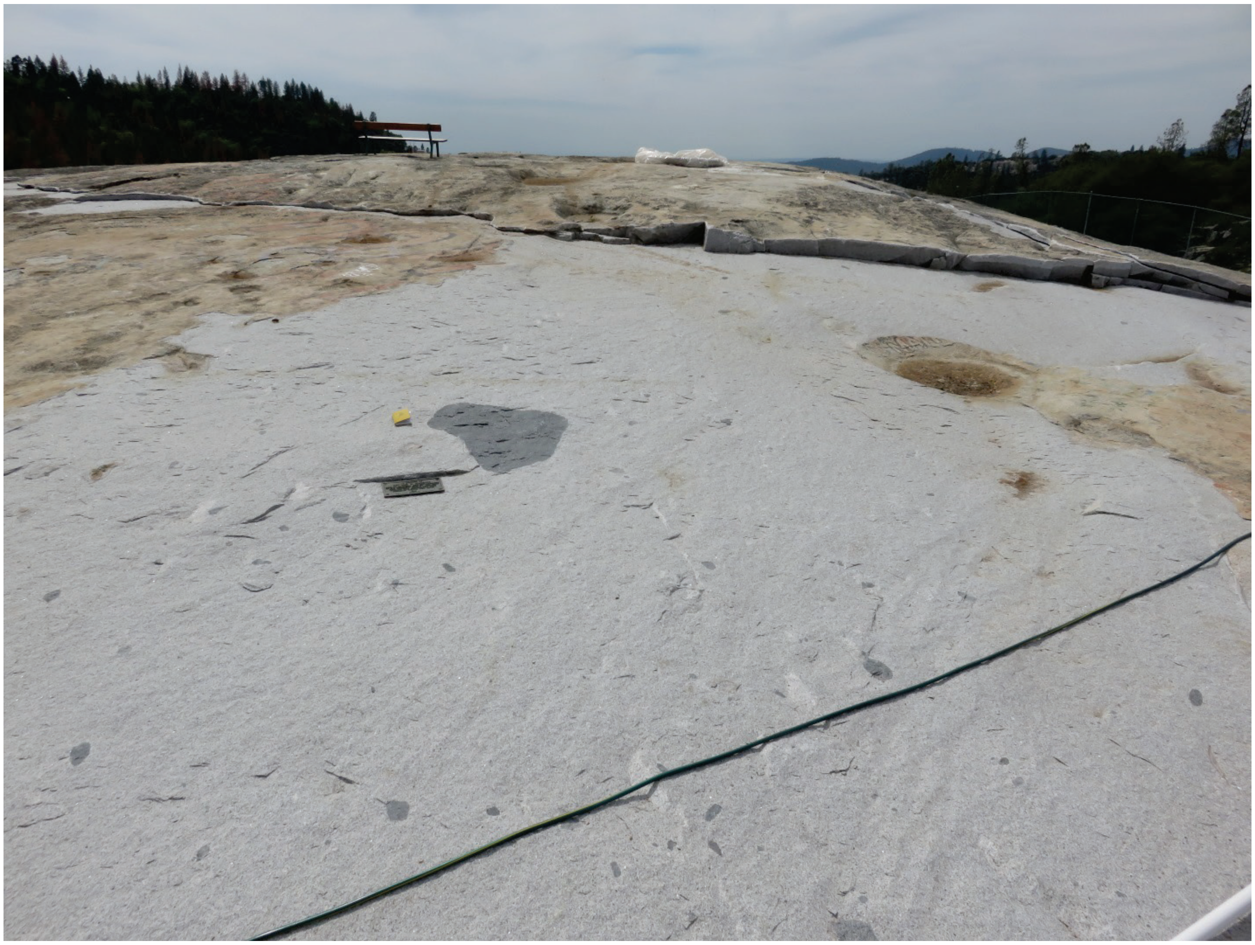

Supplementary Figure 2. Exposed fracture surface beneath ruptured 2014 exfoliation sheet. The overlying sheet has been removed thereby revealing both the partially-detached sheet surface (foreground; indicated by pockets of orange staining amongst predominantly grey rock) and a profile of decimeter-thick ruptured sheets (background). The rock surface displays shingle-like structures (centimeter-scale steps in light and dark grey rock in middle of photo) that forms a roughly arcuate pattern running north to southeast. View is to the west. Yellow field notebook for scale. A black instrument cable runs across the bottom right side of the photo.

Supplementary Figure 3

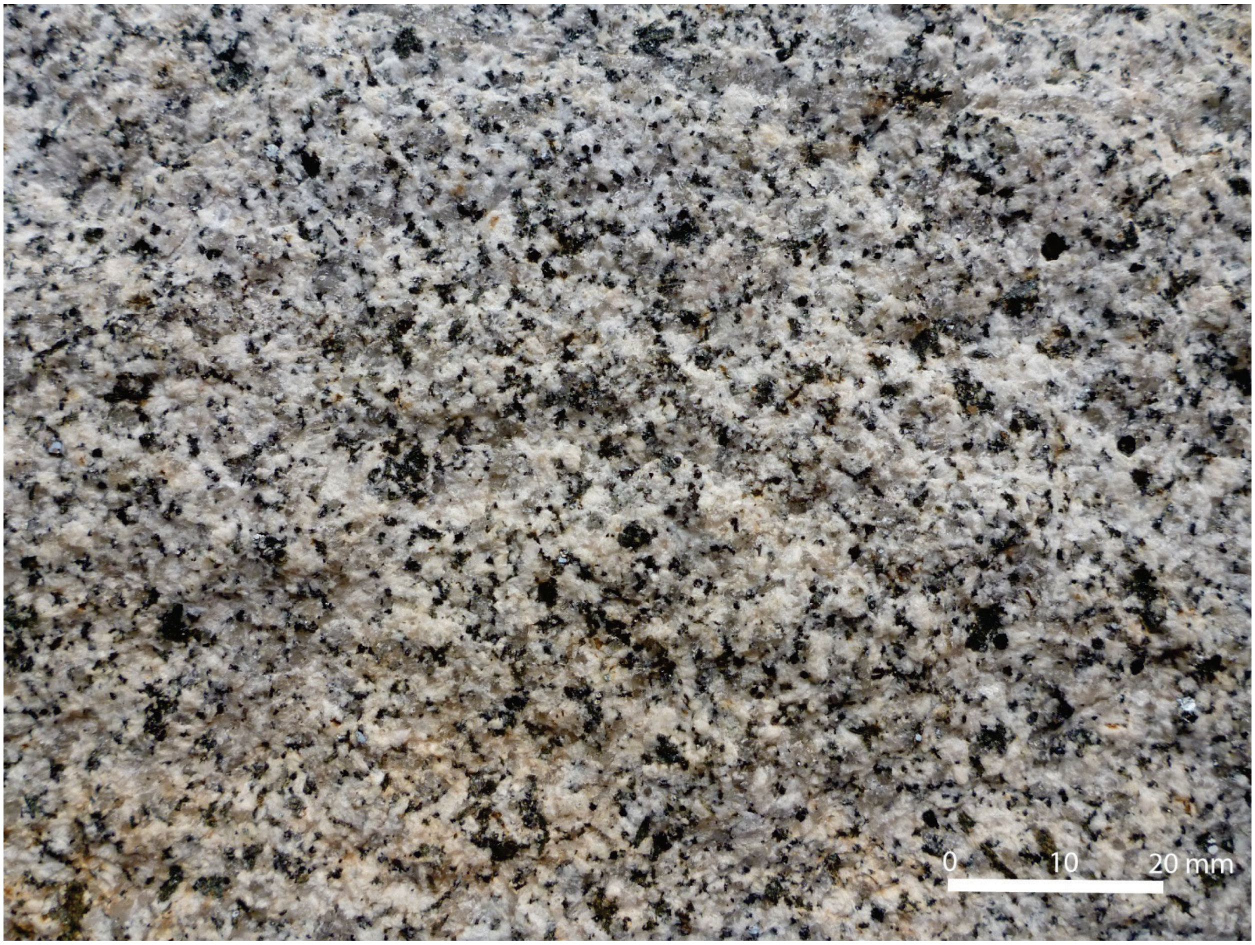

Supplementary Figure 3. Close-range photograph of the Mesozoic granodiorite forming Twain Harte Dome. The average grain size is 1-2 mm (see scale bar in lower right corner). The rock is equigranular aside from scattered biotite clots and plagioclase crystals approximately 5 mm in diameter. The rock consists of approximately 30 volume% smoky quartz, 15 volume% biotite, 5 volume% hornblende, 50 volume% feldspar (with plagioclase dominant over potassium-feldspar by 3:1), and trace titanite. Mafic minerals are largely fresh with little evidence of chloritic or other alteration visible in hand sample.

Supplementary Figure 4

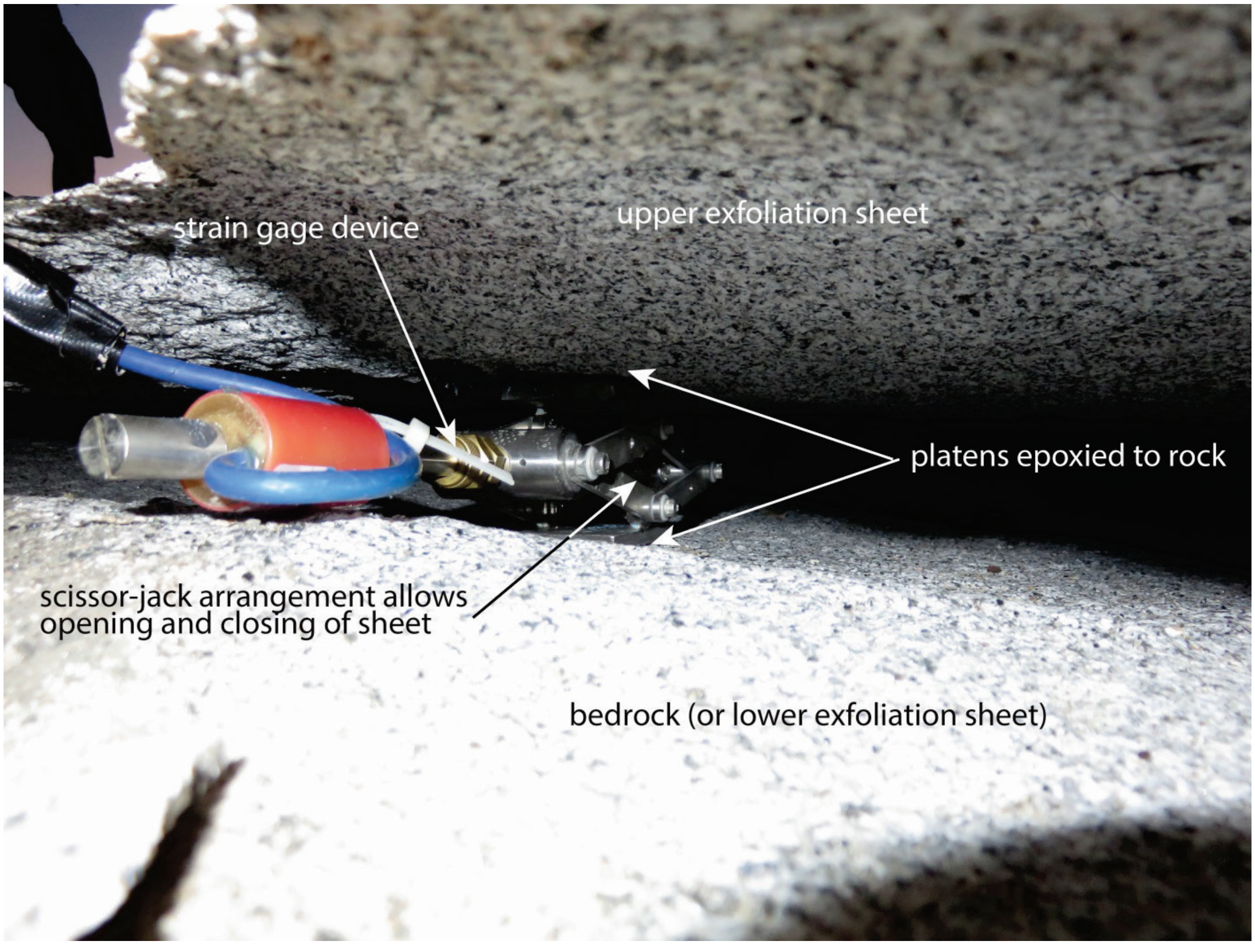

Supplementary Figure 4. Installation photograph of a crackmeter used to measure exfoliation sheet deformation. The strain gage device is calibrated to match the opening and closing of the scissor-jack arrangement.

Supplementary Figure 5

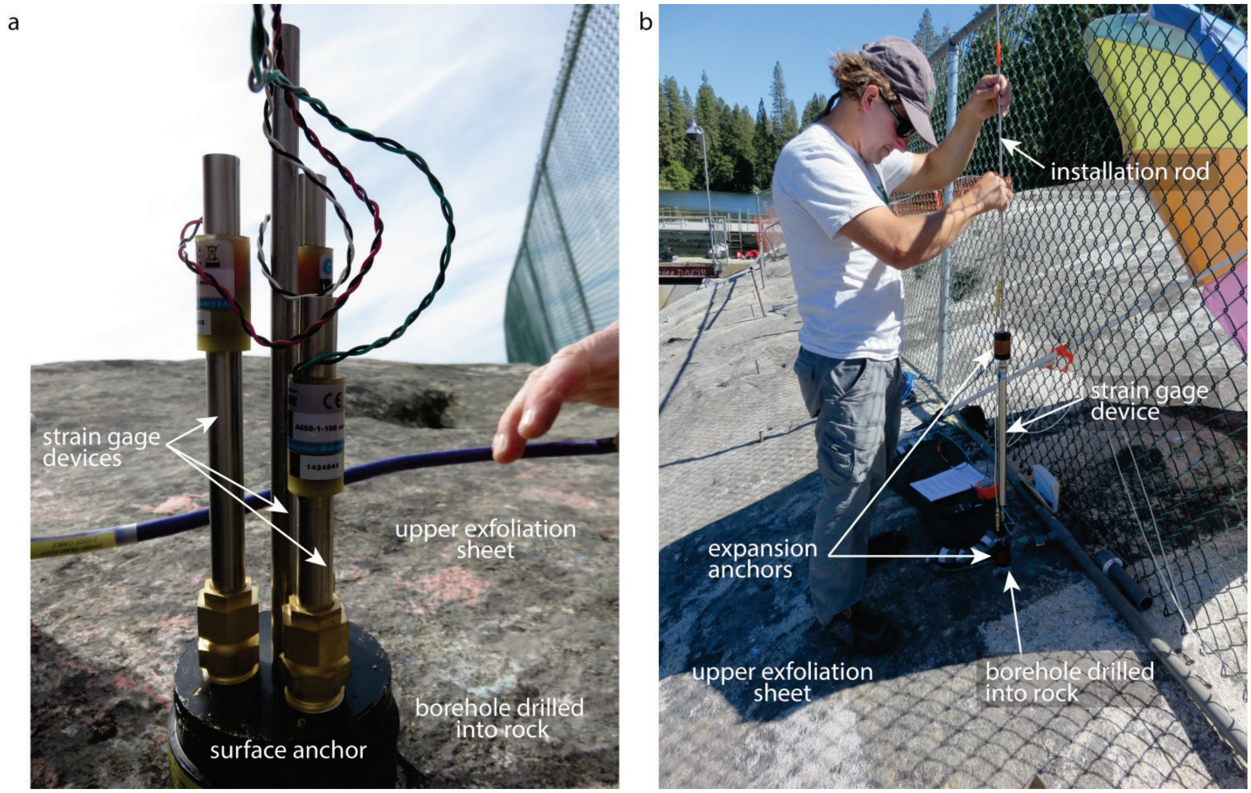

Supplementary Figure 5. Installation photographs of extensometers used to measure exfoliation sheet deformation. **a**, The above ground part of the installation consists of the ends of the strain gage devices which are anchored to the upper exfoliation sheet surface. **b**, Installation of a similar extensometer used elsewhere at Twain Harte (data not reported here). The below ground part of extensometer installations consist of hydraulic anchors used to fix the extensometer in position at depth across exfoliation fractures.

Supplementary Figure 6

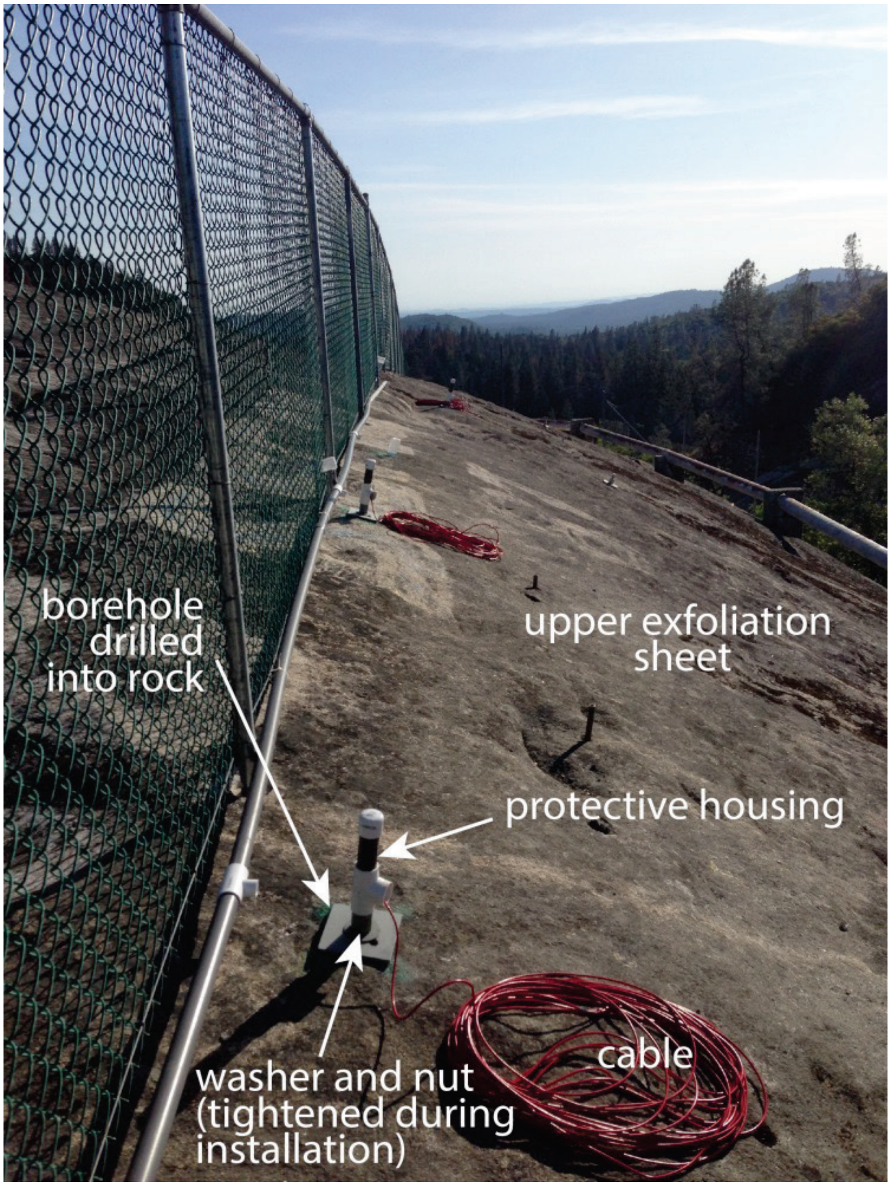

Supplementary Figure 6. Installation photograph of rockbolts used to measure exfoliation sheet uplift forces. A strain gage is inserted into a steel rod and the bottom end of the rod is grouted to the rock at depth after borehole drilling. The top of the rod is tightened via a mounting plate (washer) and nut to be snug to the exfoliation sheet rock surface. Strains are measured across the steel rod as exfoliation sheets deform inwards and outwards. Strains are converted to forces through linear elastic constants for the steel rod determined prior to installation.
