# Supplementary material for: Thermal influences on spontaneous rock dome exfoliation
Source: Nat Commun. 2018 Feb 22;9:762. doi: 10.1038/s41467-017-02728-1 (PMC5823905; doi:10.1038/s41467-017-02728-1)
Supplement: Supplementary file 2 — Description of Additional Supplementary Files [file 41467_2017_2728_MOESM2_ESM.pdf]

Description of Additional Supplementary Files

File Name: Supplementary Data 1
Description: **Time series data file for deformation, uplift force, environmental monitoring, and acoustic emissions instrumentation for the presented data at Twain Harte Dome.** Deformation data is provided for the crackmeters (CM1, CM2, CMC) and the extensometer (EX1). Uplift force data is provided for the rockbolts (RB1, RB2, RBC). Environmental monitoring data is provided for near-surface rock temperature (RT), air temperature (AT), air relative humidity (RH) and rock temperature at two depths (RT). Acoustic emissions data is provided for the six acoustic emissions sensors (AE1-6).

File Name: Supplementary Movie 1
Description: **Video of energetic exfoliation of Twain Harte Dome on 6 August 2014.** Video captures the critical fracture uplift of a 4 cm thick, 10 m diameter exfoliation sheet on the southwest side of the dome shortly after 16:00 (PST) (17:00 local time). Fragments several centimeters in longest dimension were ejected more than 4 m into the air and rock dust was ejected from all sides of the newly detached sheet. The fracture event followed several minutes of audible cracking with small fragment ejecta, and was secondary to a major fracture event that occurred several minutes prior.
